# Early Postoperative Echocardiographic and Natriuretic Peptide Changes Following Anatomical Lung Resection: A Single-Center Cohort Study

**DOI:** 10.3390/jcm15187273

**Published:** 2026-09-18

**Authors:** Mădălina Butas, Stelian Adrian Ritiu, Sonia Elena Popovici, Gabriel Veniamin Cozma, Vasile Gaborean, Daniel Goje, Iulia Najette Crintea, Alina Ramona Buzatu, Tudor Rareș Olariu, Marilena Dinuti

**Affiliations:** 1 Faculty of Medicine, “Victor Babes” University of Medicine and Pharmacy Timisoara, 300041 Timisoara, Romania; madalina.moise@umft.ro (M.B.); rolariu@umft.ro (T.R.O.);; 2 Municipal Clinical Emergency Hospital Timisoara, 300040 Timisoara, Romania; 3 Doctoral School, “Victor Babes” University of Medicine and Pharmacy Timisoara, Eftimie Murgu Square 2, 300041 Timisoara, Romania; 4 Anaesthesia and Intensive Care Research Center (CCATITM), “Victor Babes” University of Medicine and Pharmacy Timisoara, 300041 Timisoara, Romania; 5 Discipline of Surgical Semiology I and Thoracic Surgery, Department of Surgery I, “Victor Babes” University of Medicine and Pharmacy Timisoara, 300041 Timisoara, Romania; 6 Thoracic Surgery Research Center, “Victor Babes” University of Medicine and Pharmacy Timisoara, Eftimie Murgu Square No. 2, 300041 Timisoara, Romania; 7 Advanced Research Center for Cardiovascular Pathology and Haemostaseology, Department of Internal Medicine I-Medical Semiology I, “Victor Babes” University of Medicine and Pharmacy Timisoara, 300041 Timisoara, Romania; 8 Department of Surgery I, Faculty of Medicine, “Victor Babes” University of Medicine and Pharmacy Timisoara, 300041 Timișoara, Romania; 9 Discipline of Biochemistry, Department of Biochemistry and Pharmacology, Faculty of Medicine, “Victor Babes” University of Medicine and Pharmacy Timisoara, Eftimie Murgu Square 2, 300041 Timisoara, Romania; 10 Discipline of Parasitology, Department of Infectious Disease, “Victor Babes” University of Medicine and Pharmacy Timisoara, 300041 Timisoara, Romania

**Keywords:** lung resection, lobectomy, thoracic surgery, echocardiography, perioperative echocardiography, right ventricular function, pulmonary artery pressure, postoperative complications, RV–PA coupling, natriuretic peptides

## Abstract

**Background/Objectives:** Anatomical lung resection removes part of the pulmonary vascular bed and may acutely alter right ventricular loading conditions. The magnitude and the clinical significance of these early changes remain incompletely characterized, and it is unclear whether early echocardiographic changes relate to postoperative complications. This study aimed primarily to characterize the perioperative echocardiographic response to lung resection, while associations with any postoperative complication and with a cardiopulmonary composite outcome were evaluated as a secondary, exploratory analysis. **Methods:** This prospective, single-center observational study included 40 adult patients undergoing lung resection for suspected or confirmed lung cancer. Transthoracic echocardiography was performed preoperatively and on the first postoperative day, and N-terminal pro–B-type natriuretic peptide (NT-proBNP) was measured preoperatively and at 24 h. Assessed parameters included left ventricular ejection fraction (LVEF), tricuspid annular plane systolic excursion (TAPSE), systolic pulmonary artery pressure (PASP), peak tricuspid regurgitation velocity (TRV), the TAPSE/PASP coupling ratio and NT-proBNP. The primary outcome was the paired perioperative change in those measurements. Paired comparisons used the Wilcoxon signed-rank test with matched-pairs rank-biserial correlations and bootstrap confidence intervals; exploratory comparisons used Mann–Whitney U tests and areas under the receiver operating characteristic curve (AUC) with bootstrap confidence intervals. Multivariable modeling was not performed. **Results:** All six parameters changed significantly between the preoperative and postoperative assessments. PASP increased by a median of 4.0 mmHg (*p* < 0.001) and TRV max by 0.10 m/s (*p* < 0.001), while TAPSE decreased by 0.10 cm (*p* = 0.015) and LVEF by 2.0 percentage points (*p* = 0.001). The right ventricular–pulmonary arterial (RV–PA) coupling ratio declined from 0.717 to 0.526 mm/mmHg (*p* < 0.001) and NT-proBNP rose from 82.5 to 229.0 pg/mL (*p* < 0.001). Sixteen patients (40.0%) developed any complication and seven (17.5%) developed a cardiopulmonary complication. No echocardiographic parameter showed statistically supported discrimination for either outcome; all corresponding confidence intervals for the AUC included 0.50. NT-proBNP measured at 24 h and its perioperative change showed nominal associations with complication status, whereas the preoperative concentration did not. All recorded complications were documented before the 24-h blood draw. **Conclusions:** Anatomical lung resection was followed by statistically detectable changes in measured echocardiographic indices by the first postoperative day. The cohort-level increase in pulmonary pressures and the decline in RV–PA coupling were the most clearly supported findings, whereas the small changes in TAPSE and LVEF are of uncertain biological magnitude. No echocardiographic parameter showed statistically supported discrimination between patients who did and did not develop complications, as estimates were imprecise, with wide confidence intervals. The association with 24-h NT-proBNP was post-event and therefore cannot be interpreted as evidence of predictive value.

## 1. Introduction

Lung cancer remains the leading cause of cancer-related mortality worldwide, accounting for approximately 1.8 million deaths annually and representing nearly 18% of all cancer deaths [1]. The majority of cases are diagnosed as non-small-cell lung cancer (NSCLC), for which surgical resection represents the cornerstone of curative-intent treatment in patients with localized disease [2]. Anatomical lobectomy—the surgical removal of one pulmonary lobe—is the recommended standard of care for stages I–II NSCLC, offering the optimal balance between oncological radicality and preservation of pulmonary function [3]. However, beyond the loss of functioning parenchyma, resection removes a portion of the pulmonary vascular bed, and the remaining circulation must accommodate the entire cardiac output. The resulting increase in pulmonary vascular resistance imposes an acute load on the right ventricle, which is thin-walled and comparatively intolerant of abrupt afterload elevation [3,4].

Postoperative cardiopulmonary complications, including atrial fibrillation, pulmonary edema, respiratory failure, and pulmonary embolism, occur in a clinically relevant proportion of patients (10–40%, depending on the population and definitions used), contributing to prolonged hospital stay, increased healthcare utilization, and increased mortality [5,6]. Whether these events are preceded, accompanied or followed by measurable changes in right heart function is of practical interest, since transthoracic echocardiography is non-invasive, widely available and repeatable at the bedside.

Several echocardiographic parameters have been proposed for the assessment of right ventricular performance in this setting. Tricuspid annular plane systolic excursion (TAPSE) quantifies longitudinal systolic excursion of the tricuspid annulus, while Systolic pulmonary artery pressure (PASP), estimated from the peak tricuspid regurgitant velocity, reflects the load against which the ventricle contracts [7,8]. More recently, the ratio between the two has been proposed as a non-invasive surrogate of right-ventricular-to-pulmonary-arterial coupling, on the basis that contractility and afterload should be interpreted jointly rather than in isolation [9]. Reduced coupling has been associated with adverse outcomes in heart failure and in pulmonary hypertension, and has attracted interest in surgical populations [10].

Natriuretic peptides provide a complementary, load-dependent measure of ventricular wall stress. N-terminal pro–B-type natriuretic peptide (NT-proBNP) rises in response to volume and pressure loading of either ventricle and has been studied as a perioperative risk marker in non-cardiac surgery [11,12]. Its behavior in the immediate period after lung resection and its relationship to concurrent echocardiographic change are less well described.

Existing studies in this field are heterogeneous with respect to the timing of imaging, the parameters reported and the outcomes considered, and many focus on prediction in samples that are small relative to the number of events observed. The present study was therefore designed with a primarily descriptive objective: to characterize, in a consecutive surgical cohort, the paired changes in echocardiographic parameters and NT-proBNP occurring between the preoperative assessment and the first postoperative day. As a secondary and explicitly exploratory objective, we examined whether these changes were associated with postoperative complications, recognizing that the number of events available would not support inferential conclusions about prediction [13,14].

## 2. Materials and Methods

### 2.1. Study Design and Patient Population

This was a prospective, single-center observational cohort study conducted at Municipal Clinical Emergency Hospital, Timisoara, Romania, between July 2025 and December 2025. The study included adult patients consecutively diagnosed with suspected or confirmed lung cancer who were scheduled to undergo elective lung resection. The study population consisted predominantly of patients undergoing anatomical lobectomy (92.5%), with a small number of bilobectomies (7.5%).

Inclusion criteria were: age ≥ 18 years, suspected or confirmed lung cancer, indication for elective lung resection with curative intent, and provision of written informed consent. Exclusion criteria included prior cardiac surgery, pre-existing severe valvular heart disease, inadequate echocardiographic window precluding reliable assessment, and emergency surgical procedures.

The study was conducted in accordance with the Declaration of Helsinki, approved by the Institutional Review Board of the Municipal Clinical Emergency Hospital Timisoara (approval number 5582/8 October 2024) and by the Institutional Ethics Committee for Scientific Research of the “Victor Babeș” University of Medicine and Pharmacy Timisoara (approval number 91/1 October 2023_rev2025). All participants provided written informed consent prior to enrollment.

**Figure 1:**
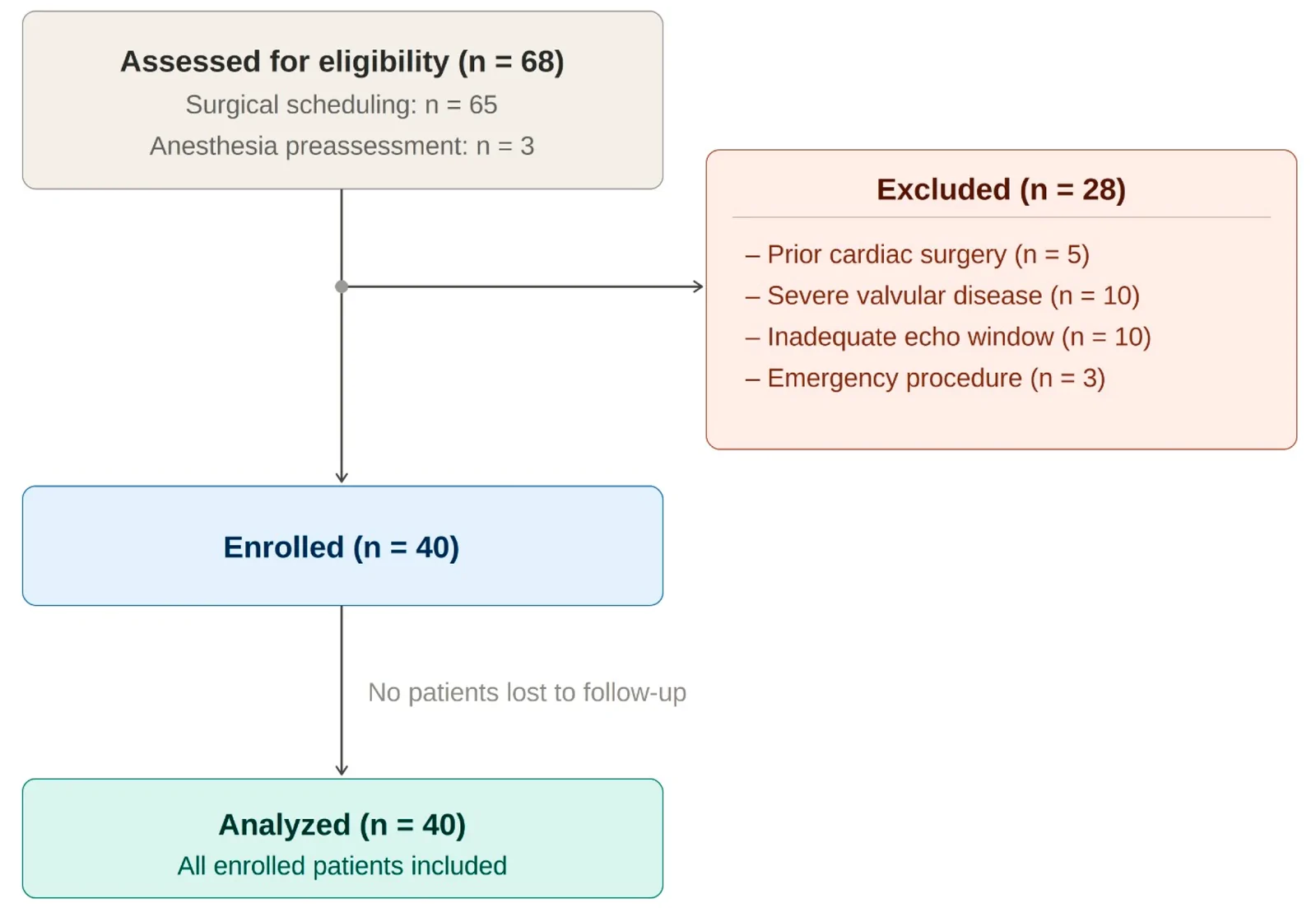
STROBE flow diagram illustrating the patient selection process. Of 68 patients assessed for eligibility, 28 were excluded and 40 were enrolled and included in the final analysis.

The patient selection process is illustrated in Figure 1. Of 68 patients assessed for eligibility—65 at the point of surgical scheduling and 3 during anesthesia preassessment—28 were excluded prior to enrolment: 5 had a history of prior cardiac surgery, 10 had pre-existing severe valvular heart disease, 10 had an inadequate echocardiographic window precluding reliable assessment, and 3 were scheduled for emergency surgical procedures. The remaining 40 patients were enrolled and all completed the study protocol; both preoperative and postoperative echocardiographic assessments were obtained for the entire cohort, with no patients lost to follow-up or excluded from analysis.

Given the exploratory and pilot nature of the study, no formal a priori sample size calculation was performed; the cohort comprised all eligible patients consecutively within the study period.

### 2.2. Surgical Procedure and Perioperative Management

All patients underwent a standardized preoperative cardiopulmonary assessment protocol, including pulmonary function testing (spirometry and diffusing capacity of the lungs for carbon monoxide (DLCO)), resting 12-lead electrocardiography, transthoracic echocardiography, cardiac biomarkers (troponin I and NT-proBNP), contrast-enhanced computed tomography (CT) of the thorax, and cardiology consultation when clinically indicated.

All patients underwent elective anatomical lung resection performed by the same surgical team under general anesthesia with one-lung ventilation using a double-lumen endotracheal tube. Anesthetic management followed institutional protocols and included standard induction and maintenance techniques, with intraoperative monitoring according to current guidelines.

Surgical procedures were performed using either open thoracotomy (in 31 patients) or video-assisted thoracoscopic surgery (VATS) (for 9 patients), depending on tumor characteristics and surgeons’ preferences. The extent of resection consisted predominantly of anatomical lobectomy, with a small number of bilobectomies performed when clinically indicated. Resections involved the right lung in 25 patients and the left lung in 15. No pneumonectomy was performed.

Perioperative management followed a standardized institutional care pathway, with all patients admitted postoperatively to the intensive care unit for continuous monitoring. Early mobilization, optimized pain control, and respiratory physiotherapy were systematically implemented during the postoperative period.

### 2.3. Echocardiographic Assessment

Transthoracic echocardiography (TTE) was performed using a commercially available ultrasound system (GE Healthcare, Chicago, IL, USA). All examinations were carried out by an experienced cardiologist trained in echocardiography, in accordance with current international recommendations.

A comprehensive echocardiographic evaluation was performed at two predefined timepoints. The preoperative assessment was conducted within 24 h prior to surgery. The postoperative assessment was performed on the first postoperative day, according to a standardized institutional protocol. The first postoperative day was chosen as a reproducible, protocol-driven point feasible within the thoracic-surgical intensive care pathway, and it is used throughout this report to describe the timing of postoperative imaging. Earlier assessment, within the first hours after surgery, would capture the immediate postoperative transition, and later or serial assessment the resolution phase; neither was within the scope of this single-timepoint design.

Left ventricular systolic function was assessed by calculating the left ventricular ejection fraction (LVEF) using the biplane Simpson method from apical two- and four-chamber views. Right ventricular systolic function was evaluated using tricuspid annular plane systolic excursion (TAPSE), measured in M-mode in the apical four-chamber view.

Pulmonary artery systolic pressure (PASP) was estimated based on the peak tricuspid regurgitation velocity (TRV) using the modified Bernoulli equation: PASP = 4 × TRV^2^ + RAP.

Right atrial pressure (RAP) was estimated according to the inferior vena cava (IVC)’s diameter and its respiratory variation, in accordance with standard echocardiographic recommendations.

Valvular function was assessed qualitatively, with tricuspid and mitral regurgitation graded according to standard clinical practice.

To evaluate perioperative changes, delta values (Δ) were calculated as the difference between postoperative and preoperative measurements.

Two constraints of the design should be noted. Examinations were performed by a single experienced operator who was not blinded to the timing of the study or to the clinical course, and no formal intra-observer or inter-observer reproducibility assessment was undertaken. In addition, although all postoperative examinations were performed on the first postoperative day, the exact interval between surgery and imaging was not recorded, and the physiological conditions at the time of imaging, including supplemental oxygen or ventilatory support, vasoactive medication, fluid balance, cardiac rhythm and analgesia, were not systematically documented. These variables were therefore unavailable for analysis, and no claim is made regarding the extent to which acute perioperative physiological effects had resolved at the time of imaging.

### 2.4. Biomarker Sampling

NT-proBNP and troponin I were measured preoperatively and at 24 h after surgery as part of routine perioperative care. The perioperative change in NT-proBNP was defined as the 24-h value minus the preoperative value. Unlike the echocardiographic assessment, biomarker sampling was fixed by protocol at 24 h; the term “24 h” is used in this report exclusively for the biomarker draw. Troponin I values are reported as part of the baseline description but were not included in the analyses presented here.

### 2.5. Outcome Definitions

The primary outcome was paired perioperative change from baseline to the postoperative assessment: postoperative day 1 for echocardiographic parameters and 24 h for NT-proBNP.

Two secondary, exploratory outcomes were defined. The first was the occurrence of any postoperative complication during the index admission. The second was a cardiopulmonary composite comprising atrial fibrillation, pulmonary edema or pulmonary embolism, defined to group events with a plausible mechanistic link to right heart and pulmonary hemodynamic changes. All recorded complications were documented in the clinical notes prior to the 24-h biomarker sampling; the precise time of onset of individual events was not recorded and could not be reconstructed.

### 2.6. Statistical Analysis

#### 2.6.1. Software and Reproducibility

All statistical analyses were performed using Python (version 3.12.1). The following libraries were used: pandas (version 3.0.1), scipy (version 1.17.0), statsmodels (version 0.14.6), pingouin (version 0.6.1), scikit-learn (version 1.8.0), matplotlib (version 3.10.8) and seaborn (version 0.13.2).

Continuous variables are reported as median [interquartile range] or as mean ± standard deviation where the distribution was approximately symmetric with categorical variables as counts (percentages). All tests were two-sided with an alpha of 0.05.

The analysis was structured around one primary objective and one secondary objective. The paired perioperative comparisons constitute the primary descriptive and inferential analysis. All analyses involving postoperative complications, including group comparisons, discrimination and subgroup comparisons, are secondary, exploratory, and hypothesis-generating. Nominal, unadjusted *p*-values are reported; because eight predictors were examined against two outcomes and four parameters across three subgroup contrasts, no correction for multiplicity was applied, and isolated significant findings may reflect chance.

#### 2.6.2. Paired Comparisons

The choice of paired test was based on the distribution of the within-patient differences rather than on separate normality testing of the preoperative and postoperative measurements. The differences were examined graphically by histogram, quantile–quantile plot, and with the Shapiro–Wilk test; they departed from normality for every parameter, and the Wilcoxon signed-rank test was therefore applied uniformly (Supplementary Table S4 and Supplementary Figure S1). Although the six paired comparisons are presented as a descriptive panel rather than as independent confirmatory tests, a family-wise sensitivity check was performed across them using the Holm–Bonferroni procedure, which controls the family-wise error rate under arbitrary dependence between tests.

Effect sizes for paired comparisons are reported as the matched-pairs rank-biserial correlation, with positive values indicating predominantly higher postoperative measurements and negative values predominantly lower. Standardized mean differences were not used, as they are not the appropriate companion to a rank-based test and were not employed to infer statistical power. Confidence intervals for all effect sizes were obtained by percentile bootstrap with 1000 resamples and a fixed random seed, resampling complete pre–post pairs.

#### 2.6.3. Calculation of the RV–PA Coupling Ratio 

The right-ventricular-to-pulmonary-arterial coupling ratio was calculated as TAPSE, converted from centimeters to millimeters and divided by PASP in mmHg, and is reported in mm/mmHg. The ratio was analyzed exclusively as a continuous variable. Patients were not dichotomized into preserved and impaired coupling categories, as no validated threshold exists for the early postoperative setting; the distribution of the cohort relative to two reference values reported in the literature is provided for descriptive context in the Supplementary Materials, without formation of groups or associated testing.

#### 2.6.4. Exploratory Complication Analyses

Associations between the perioperative changes and each outcome were assessed by Mann–Whitney U tests, with rank-biserial correlations and bootstrap confidence intervals as mentioned above, stratified within group. Discrimination was summarized by the AUC with percentile bootstrap of 95% confidence intervals using 1000 resamples and a fixed seed. The positive class was defined as occurrence of the complication and each predictor was entered in its recorded direction without sign inversion; values below 0.50 therefore indicate that lower predictor values accompanied complications. Because thresholds derived from and evaluated in the same small sample are unstable and optimistically biased, no cut-offs, sensitivities or specificities were derived.

Given only 16 and 7 outcome events, adjusted multivariable estimates were considered insufficiently stable and were not fitted. All complication analyses are therefore unadjusted.

#### 2.6.5. Subgroup Analyses

Three predefined exploratory subgroup contrasts were examined: surgical approach, open versus video-assisted; resection side, right versus left; and lobe of resection. For the lobe analysis, upper lobe resections, right or left, were compared with lower lobe resections; middle lobe resections were reported descriptively and bilobectomies were excluded as a different operation. A directional expectation was specified in advance for this contrast only: because the lower lobes receive a greater share of pulmonary perfusion, larger increases in PASP and TRV max were anticipated after lower lobe resection, and these two parameters were designated as primary with TAPSE and LVEF as secondary. The comparisons by surgical approach and by resection side were examined without a prespecified directional hypothesis. All parameters are reported regardless of significance and all tests were two-sided.

#### 2.6.6. Sensitivity Analysis

Achieved or observed power was not computed, since power calculated from an observed effect size is a deterministic function of the *p*-value and provides no additional information. For context on the design, the magnitude of effect that the achieved sample size would have been able to detect is reported in the Supplementary Materials (Supplementary Table S6). These are design quantities, derived from the sample size, group sizes, significance level and a conventional power target, and they are not used to interpret individual observed results; after the data are observed, precision is conveyed by the effect estimates and their confidence intervals, which are reported throughout.

## 3. Results

### 3.1. Patient Characteristics

Forty patients were included in the analysis. The cohort had a mean age of 59.4 ± 12.6 years and a substantial comorbidity burden (median Charlson Comorbidity Index of 4 [IQR: 2–6]).

Open thoracotomy was the predominant surgical approach (77.5%), and lobectomy was the most frequent resection type (92.50%); the cohort also included bilobectomy (7.5%).

Thirty-eight of the 40 patients were classified as American Society of Anesthesiologists (ASA III), with one patient each in classes II and IV.

Baseline demographic, functional and oncological characteristics are presented in Table 1.

The seven cardiopulmonary complications comprised atrial fibrillation in four patients, pulmonary edema in two and pulmonary embolism in one.

### 3.2. Perioperative Changes in Echocardiographic Parameters and NT-proBNP

**Figure 2:**
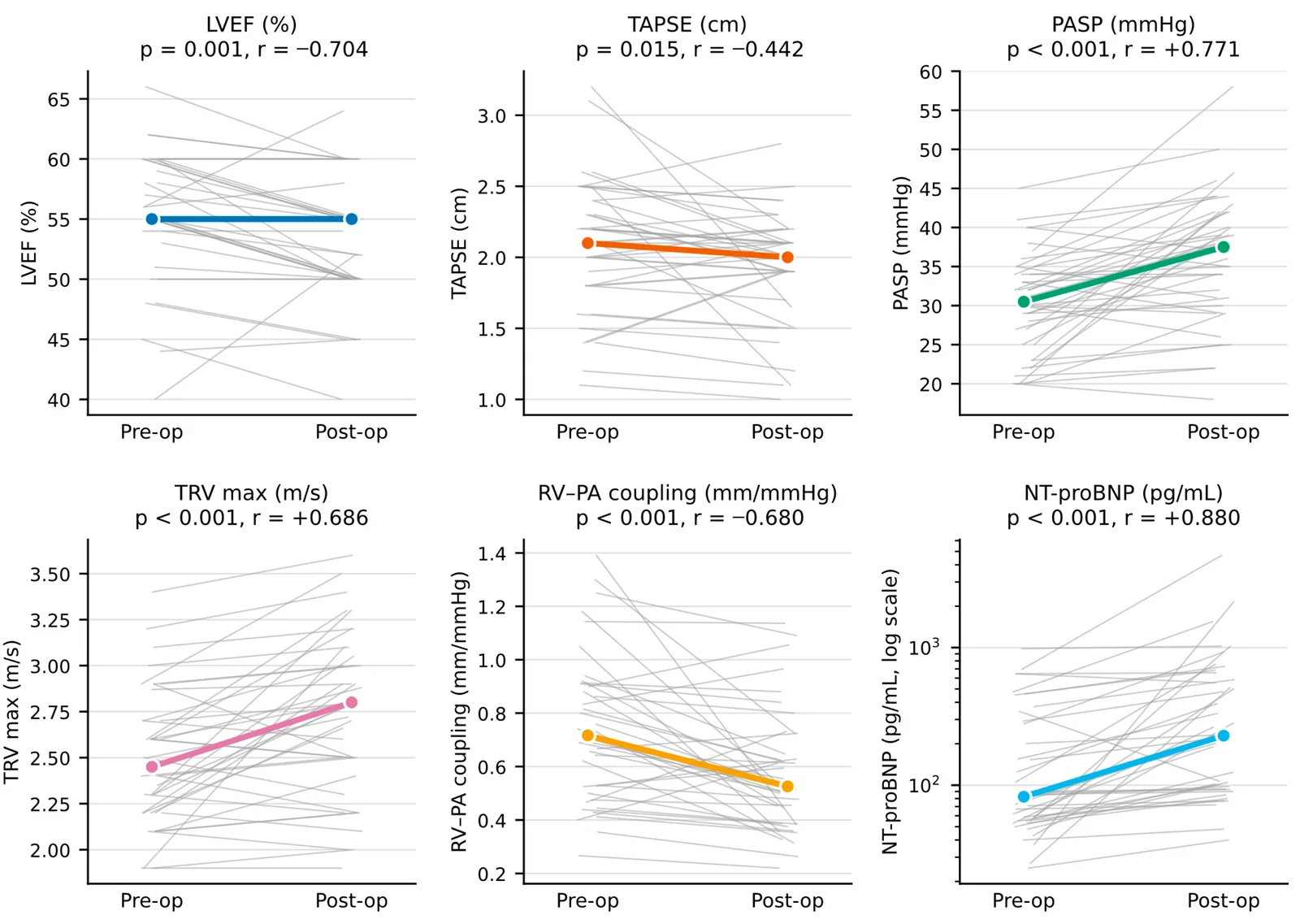
Paired changes.

All six parameters changed significantly between the preoperative assessment and the first postoperative day (Table 2 and Figure 2). Pulmonary pressures rose consistently: PASP increased by a median of 4.0 mmHg, from 30.5 [25.0 to 34.2] to 37.5 [30.8 to 42.0] mmHg (*p* < 0.001; r = +0.771), and TRV max by 0.10 m/s (*p* < 0.001; r = +0.686). Right ventricular longitudinal function declined modestly, with TAPSE falling by a median of 0.10 cm (*p* = 0.015; r = −0.442), and LVEF decreased by a median of 2.0 percentage points (*p* = 0.001; r = −0.704).

Consistent with the combination of increased afterload and reduced longitudinal function, the RV–PA coupling ratio declined from 0.717 [0.519 to 0.902] to 0.526 [0.386 to 0.666] mm/mmHg, a median change of −0.108 [−0.256 to −0.032] (*p* < 0.001; r = −0.680). The largest single effect was the natriuretic peptide response: NT-proBNP rose from 82.5 [55.0 to 282.2] to 229.0 [97.0 to 602.0] pg/mL (*p* < 0.001; r = +0.880). The bootstrap confidence intervals for all six effect sizes excluded zero, indicating a statistically supported cohort-level shift in the corresponding direction. Individual patients nevertheless varied considerably, including in the direction of change: the perioperative change in PASP ranged from −4 to +22 mmHg, in TRV max from −0.40 to +1.20 m/s, in LVEF from −10 to +12 percentage points and in TAPSE from −1.55 to +0.50 cm. The six paired comparisons form a single interdependent panel and their *p*-values are reported as nominal. As a sensitivity check, the Holm–Bonferroni procedure was applied across the six comparisons; all six remained statistically significant after adjustment, with the largest adjusted value being that for TAPSE (adjusted *p* = 0.015).

Two patients showed within-patient TAPSE reductions exceeding 1.0 cm. A unit and sign audit of the TAPSE variables confirmed that both source measurements lay within the plausible centimeter range, that the difference reproduced exactly as the postoperative-minus-preoperative value, and that no unit conversion or sign inversion had occurred. These are therefore genuine observed changes rather than data-entry artifacts and are reported as recorded.

### 3.3. Exploratory Associations with Postoperative Complications

Sixteen patients (40.0%) developed any postoperative complication and seven (17.5%) developed a cardiopulmonary complication. No echocardiographic parameter showed a statistically supported association with either outcome (Table 3). All confidence intervals for the rank-biserial correlation included zero, and all confidence intervals for the area under the receiver operating characteristic curve (AUC) included 0.50, for both the any-complication outcome (AUC range, 0.389 to 0.503) and the cardiopulmonary outcome (AUC range, 0.316 to 0.494). The RV–PA coupling ratio showed no statistically supported association with either outcome despite its significant perioperative decline (change in coupling, *p* = 0.629 and *p* = 0.972 respectively).

The 24-h NT-proBNP concentration and its perioperative change showed nominal associations with complication status. For the cardiopulmonary outcome, the 24-h concentration was higher in affected patients, with 929.0 [418.5 to 1592.5] versus 190.0 [93.0 to 501.0] pg/mL (*p* = 0.004; AUC 0.851, 95% CI 0.681 to 0.983), and the perioperative change was likewise higher (*p* = 0.019), although the bootstrap confidence interval for the corresponding AUC included 0.50 (0.788, 95% CI 0.461 to 0.989). The same direction was observed for any complication, for both the 24-h concentration (473.0 [194.2 to 1024.0] versus 157.0 [89.8 to 483.0] pg/mL; *p* = 0.015; AUC 0.732, 95% CI 0.544 to 0.875) and the perioperative change (*p* = 0.047; AUC 0.689, 95% CI 0.503 to 0.854). The preoperative concentration was not associated with either outcome (*p* = 0.571 and *p* = 0.749; AUC, 0.555 and 0.459). These exploratory estimates have wide confidence intervals and are unadjusted for multiplicity.

All recorded complications were documented before the 24-h blood draw, so the postoperative concentration was measured after the onset of the events with which it is associated. The observed association is therefore post-event and cannot establish prediction or temporal ordering. Because eight predictors were screened against two outcomes without correction for multiplicity, these nominal *p*-values are hypothesis-generating.

### 3.4. Exploratory Subgroup Analyses

Perioperative changes were compared across three predefined subgroups, in each case using Mann–Whitney U tests without adjustment. No significant differences were observed by surgical approach, comparing 31 open with nine video-assisted procedures, with a median change in PASP of 4.0 versus 10.0 mmHg (*p* = 0.548) and no difference in TAPSE (*p* = 0.230) (Supplementary Table S1). Likewise, no significant differences were observed by resection side, comparing 25 right-sided with 15 left-sided resections, with a median change in PASP of 3.0 versus 5.0 mmHg (*p* = 0.287) and no difference in TRV max (*p* = 0.447) (Supplementary Table S2). A larger pulmonary hemodynamic response after right-sided resection would be physiologically plausible, given the greater share of the vascular bed carried by the right lung, but this expectation was not prespecified and was not observed.

By lobe of resection, 21 upper lobe and 14 lower lobe resections were compared directly, with two middle lobe resections reported descriptively and three bilobectomies excluded. Both primary parameters moved in the direction expected from the greater perfusion of the lower lobes, but neither difference was statistically supported: the change in PASP was a median of 2.0 mmHg larger after lower lobe resection, with 5.0 [2.2 to 13.8] versus 3.0 [0.0 to 10.0] mmHg (*p* = 0.389; r = +0.177, 95% CI −0.224 to 0.555), and the change in TRV max was 0.14 m/s larger, with 0.24 [0.10 to 0.58] versus 0.10 [−0.10 to 0.46] m/s (*p* = 0.285; r = +0.218, 95% CI −0.160 to 0.616). The secondary parameters showed no difference (Supplementary Table S3 and Supplementary Figure S2).

All subgroup comparisons involve small groups, have wide confidence intervals, and are descriptive and hypothesis-generating.

## 4. Discussion

This study characterized the perioperative echocardiographic response to lung resection for suspected or confirmed lung cancer and evaluated its association with postoperative cardiopulmonary complications. The cohort consisted predominantly of lobectomies (92.5%), with a small number of bilobectomies (7.5%).

In this cohort of 40 patients undergoing anatomical lung resection, all six measured parameters changed significantly between the preoperative assessment and the first postoperative day. The direction of change was physiologically coherent: pulmonary pressures and tricuspid regurgitant velocity rose; right ventricular longitudinal function and left ventricular ejection fraction fell modestly; the ratio between contractility and afterload declined, and NT-proBNP rose substantially [15]. None of these changes showed statistically supported differences between patients who developed complications and those who did not, in a cohort whose size permitted detection of only large differences.

### 4.1. Magnitude and Interpretation of the Observed Changes

The most pronounced echocardiographic changes were those relating to pulmonary pressure. The increases in PASP of 4.0 mmHg and in TRV max of 0.10 m/s were supported at the cohort level, with rank-biserial correlations of +0.771 and +0.686 and confidence intervals well clear of zero. This pattern is compatible with the expected redistribution of the entire cardiac output through a reduced vascular bed [3,4].

The changes in TAPSE and LVEF, by contrast, were small in absolute terms: a median reduction of 0.10 cm and of 2.0 percentage points respectively. Although both reached statistical significance, their clinical interpretation requires caution [16]. All examinations were performed by a single operator without a formal reproducibility assessment, and changes in this order approach the range of measurement variability reported for these parameters [7,8]. Measurement variability therefore cannot be fully separated from small biological change, and we do not interpret these two findings as evidence of clinically meaningful ventricular dysfunction. The cohort-level directional shift was statistically supported; however, in the absence of reproducibility assessment, systematic measurement effects cannot be excluded.

### 4.2. RV–PA Coupling

The TAPSE/PASP ratio declined significantly, from 0.717 to 0.526 mm/mmHg. Because the ratio combines a contractility term and an afterload term, it captures in a single quantity the divergence between the two components described above, and its decline indicates that the increase in load was not matched by a corresponding increase in longitudinal function [17]. This is consistent with the concept of acute right-ventricular-to-pulmonary-arterial uncoupling described in other settings [9,10,18].

The ratio did not, however, show statistically supported discrimination between patients who developed complications and those who did not; the confidence intervals for the corresponding AUC included 0.50. This contrasts with reports in which reduced coupling has been associated with adverse outcomes [19,20,21,22]. Two considerations are relevant. First, those studies generally examined populations with a higher prevalence of established right ventricular dysfunction, in whom the range of the ratio is wider and its prognostic gradient correspondingly steeper. Second, the estimates obtained here are imprecise, and the absence of a statistically supported association in a cohort of this size should not be read as evidence against one.

We deliberately analyzed the ratio as a continuous variable and did not classify patients as having preserved or impaired coupling. Published reference values for the ratio differ substantially, and no threshold has been validated for the early postoperative setting; applying different published values to the present cohort would classify the same patients in materially different ways. Reporting the ratio continuously avoids an arbitrary dichotomy and makes fuller use of the available information.

### 4.3. Complications and the Limits of Inference

No echocardiographic parameter showed statistically supported discrimination for either outcome. Every confidence interval for the AUC included 0.50, and every confidence interval for the rank-biserial correlation included zero. These results are therefore inconclusive rather than negative, and they do not support the conclusion that early echocardiographic change is unrelated to postoperative complications. Values below 0.50 indicate that lower measurements accompanied complications, in a direction opposite to that assumed; given the width of the intervals, they are not interpreted as biological evidence in either direction.

This distinction is not merely semantic. A study of this size can establish that a change occurs, which is the primary finding reported here, without being able to establish whether that change carries prognostic information. The estimates reported here are accompanied by confidence intervals precisely so that their imprecision is visible; reporting achieved power computed from the observed effects would not have added information, since such a calculation is a restatement of the *p*-value.

The decision not to fit multivariable models follows from the same considerations [23]. With 1.75 events per parameter for the cardiopulmonary outcome, and with the resection variable near-invariant in this cohort, adjusted estimates would have been unstable and the resulting confidence intervals uninterpretably wide. Unadjusted comparisons with explicit effect sizes and confidence intervals convey the available information without implying a precision the data do not support.

### 4.4. NT-proBNP as a Post-Event Marker

NT-proBNP showed the largest perioperative change in any parameter studied, and the 24-h concentration and its perioperative change were the only measurements showing nominal associations with complication status. Their interpretation, however, is determined by timing rather than by the strength of the association. All recorded complications were documented before the 24-h blood draw, as described in the Materials and Methods section. The consequence is a limit on what can be claimed rather than an argument about mechanism. Because the biomarker was measured after the documented events, the observed association is post-event and cannot establish prediction or temporal ordering, and it does not distinguish between a biomarker rise caused by the complication, a common antecedent affecting both, and an underlying predisposition that the preoperative measurement was too imprecise to detect. The preoperative concentration was not associated with either outcome, but with seven cardiopulmonary events and a confidence interval for the corresponding AUC extending from 0.149 to 0.816, that null result cannot exclude a baseline association.

Establishing whether NT-proBNP carries predictive value would require sampling before the events occur, either preoperatively or within the first hours after surgery, in a prospectively designed cohort with documented event times.

### 4.5. Subgroup Findings

None of the three subgroups produced statistically supported differences, and all involved small groups with wide confidence intervals. Two are nevertheless worth noting. A larger pulmonary hemodynamic response after right-sided resection would be physiologically plausible, given the greater share of the vascular bed carried by the right lung, but the change in PASP was numerically larger after left-sided resection. Conversely, the anticipated larger response after lower lobe resection was directionally consistent for both primary parameters, though neither difference was statistically supported and both confidence intervals were wide. These observations are reported for completeness and as hypotheses for a larger study.

### 4.6. Limitations

Several limitations should be considered. The study was conducted at a single center in a small cohort, with a limited number of outcome events, particularly for the cardiopulmonary composite. The consequence is that only large between-group differences could have been detected, and the exploratory analyses are correspondingly imprecise; the estimates and confidence intervals reported throughout should be read with this in mind, and the magnitude of effect the design could have detected is reported as the design context in Supplementary Table S6.

Chronic and perioperative medication data were not systematically recorded in this study. In particular, beta-adrenergic blocking agents, which are commonly prescribed in this population given the high prevalence of coronary artery disease (35.0%) and arterial hypertension (57.5%), may attenuate compensatory tachycardia and reduce the magnitude of measurable echocardiographic changes in response to surgical stress. The potential confounding effect of perioperative medication on echocardiographic parameters could not be assessed and represents a priority for prospective documentation in future studies.

The predominance of open thoracotomy (77.5%) reflects institutional surgical practice during the study period and limits generalizability to centers where VATS is the standard approach. The choice of surgical technique was determined exclusively by the operating surgeons based on oncological and anatomical considerations, independent of study design. Surgical approach was examined as a predefined subgroup contrast rather than as a model covariate (Supplementary Table S1).

Postoperative echocardiography was performed at a single timepoint, on the first postoperative day. Serial assessments would more accurately capture the dynamic trajectory of post-resection cardiac remodeling but were beyond the scope of this exploratory study. Echocardiographic examinations were performed by a single operator who was not blinded, and no formal reproducibility assessment was undertaken; this is most relevant to the small changes observed in TAPSE and LVEF. Although all postoperative examinations were performed on the first postoperative day, the exact interval between surgery and imaging was not consistently documented, and the physiological conditions at the time of imaging, including ventilatory and vasoactive support, fluid balance, cardiac rhythm and analgesia, were not systematically recorded. These factors can all influence the estimation of pulmonary pressures.

All recorded complications preceded the 24-h biomarker sampling, which precludes any assessment of the predictive value of NT-proBNP in this dataset. The composite outcome of any postoperative complication is mechanistically heterogeneous; the inclusion of events such as pneumothorax and bleeding, which are not directly mediated by cardiac hemodynamics, may have attenuated potential echocardiographic signals. Future studies should therefore pre-specify a cardiopulmonary-specific primary endpoint. The cohort was almost exclusively ASA III, and while this reflects the typical profile of patients undergoing elective lung resection for lung cancer at our institution, it limits generalizability to lower- and higher-risk populations, and future studies should include patients across a broader ASA spectrum to allow adequate adjustment for baseline functional status. The cohort comprised lobectomies and bilobectomies only, so the findings do not extend to more extensive resections [24,25,26]. Finally, the complication analyses involved multiple comparisons without correction, and the associated *p*-values should be read as hypothesis-generating.

### 4.7. Implications for Further Study

The present findings define the requirements for a study capable of addressing the predictive question. Biomarker sampling would need to occur preoperatively and within the first hours after surgery, before the majority of early events; the timing of imaging and of each complication would need to be recorded prospectively; a reproducibility assessment involving independent operators would be required to interpret small changes in longitudinal function. The sample size for such a study should be derived from externally reported effect sizes and complication rates rather than from the estimates obtained here, which are imprecise and subject to the selection effects inherent in a small exploratory sample.

## 5. Conclusions

Anatomical lung resection was followed by statistically detectable changes in measured echocardiographic indices by the first postoperative day. The cohort-level increase in pulmonary pressures and the decline in the RV–PA coupling ratio were the most clearly supported findings, with effect sizes whose confidence intervals excluded zero. The accompanying changes in TAPSE and LVEF, although statistically significant, were small in absolute terms and of uncertain biological magnitude in the absence of a reproducibility assessment. No echocardiographic parameter showed statistically supported discrimination between patients who did and did not develop postoperative complications; all corresponding confidence intervals included the value expected under chance, so these results are inconclusive rather than negative. NT-proBNP was measured after the documented complications and its association with a complicated course is therefore post-event, providing no evidence of predictive value. The magnitude, reproducibility and prognostic significance of these within-patient changes remain to be established in a prospectively designed and adequately sized study.

## Figures and Tables

**Table 1:** Baseline characteristics of the study population (N = 40).

Variable	Value
Demographics	
Age (years)	59.4 ± 12.6
Female, *n* (%)	19 (47.5)
BMI (kg/m^2^)	26.6 ± 5.9
Presenting symptoms, *n* (%)	
Dyspnea	7 (17.5)
Cough	8 (20.0)
Hemoptysis	4 (10.0)
Weight loss	6 (15.0)
Chest pain	4 (10.0)
Fatigue	4 (10.0)
Comorbidity	
ASA physical status [II/III/IV]	1/38/1
Charlson Comorbidity Index	4.0 [2.0–6.0]
Arterial hypertension, *n* (%)	23 (57.5)
Diabetes mellitus, *n* (%)	10 (25.0)
COPD, *n* (%)	13 (32.5)
Asthma, *n* (%)	4 (10.0)
Coronary artery disease, *n* (%)	14 (35.0)
Smoking status, *n* (%)	
Never smoked	12 (30.0)
Former smoker	6 (15.0)
Current smoker	22 (55.0)
Pack-years (among ever-smokers, *n* = 28)	33.5 [24.0–50.0]
Laboratory and biomarkers	
Creatinine (pre-op, mg/dL)	0.9 ± 0.2
NT-proBNP (pre-op, pg/mL)	82.5 [55.0–282.2]
Troponin I (pre-op, ng/mL)	1.6 [1.5–2.1]
NT-proBNP (24 h post-op, pg/mL)	229.0 [97.0–602.0]
Troponin I (24 h post-op, ng/mL)	3.4 [2.2–5.6]
Pulmonary function	
FEV1 (% predicted)	79.5 [69.0–98.0]
DLCO (% predicted)	70.2 ± 9.8
Perioperative echocardiography	
LVEF (%)	55.0 [54.0–60.0]
TAPSE (cm)	2.10 [1.80–2.40]
PASP (mmHg)	30.5 [25.0–34.2]
TRV (m/s)	2.45 [2.20–2.70]
Postoperative echocardiography	
LVEF (%)	55.0 [50.0–55.0]
TAPSE (cm)	2.0 [1.8–2.1]
PASP (mmHg)	37.5 [30.8–42.0]
TRV (m/s)	2.80 [2.48–3.01]
Surgical details	
Open thoracotomy, *n* (%)	31 (77.5)
VATS, *n* (%)	9 (22.5)
Lobectomy, *n* (%)	37 (92.5)
Bilobectomy, *n* (%)	3 (7.5)
Right side, *n* (%)	25 (62.5)
Left side, *n* (%)	15 (37.5)
Operative time (min)	330.1 ± 63.1
Intraoperative transfusion, n (%)	5 (12.5)
Tumor characteristics	
Tumor size (max) (mm)	31.0 [21.5–42.5]
Tumor size (min) (mm)	12.5 [10.0–21.0]
Postoperative complications	
Any complication, *n* (%)	16 (40.0)
Cardiopulmonary complication, *n* (%)	7 (17.5)

Data presented as mean ± SD or median [IQR: 25th–75th percentile] for continuous variables, and n (%) for categorical variables. BMI: Body mass index; ASA: American Society of Anesthesiologists; COPD: chronic obstructive pulmonary disease; FEV1: forced expiratory volume in 1 s; DLCO: diffusing capacity of the lungs for carbon monoxide; NT-proBNP: N-terminal pro-brain natriuretic peptide; LVEF: left ventricular ejection fraction; TAPSE: tricuspid annular plane systolic excursion; PASP: systolic pulmonary artery pressure; TRV: peak tricuspid regurgitation velocity; VATS: video-assisted thoracoscopic surgery. Non-smokers were assigned 0 pack-years. Tumor sizes (maximum and minimum, respectively) denote the longest and shortest tumor diameters measured on preoperative contrast-enhanced CT.

**Table 2:** Paired perioperative changes in echocardiographic parameters and NT-proBNP (N = 40).

Parameter	Preoperative, Median [IQR]	Postoperative, Median [IQR]	Change, Median [IQR]	*p*	Rank-Biserial r [95% CI]
LVEF (%)	55.0 [54.0–60.0]	55.0 [50.0–55.0]	−2.0 [−5.0–0.0]	0.001	−0.704 [−0.992, −0.332]
TAPSE (cm)	2.10 [1.80–2.40]	2.00 [1.85–2.12]	−0.10 [−0.23–0.10]	0.015	−0.442 [−0.730, −0.124]
PASP (mmHg)	30.5 [25.0–34.2]	37.5 [30.8–42.0]	4.0 [1.0–10.0]	<0.001	+0.771 [0.529, 0.933]
TRV max (m/s)	2.45 [2.20–2.70]	2.80 [2.48–3.01]	0.10 [0.02–0.47]	<0.001	+0.686 [0.427, 0.887]
RV–PA coupl. (TAPSE/PASP, mm/mmHg)	0.717 [0.519–0.902]	0.526 [0.386–0.666]	−0.108 [−0.256, −0.032]	<0.001	−0.680 [−0.895, −0.414]
NT-proBNP (pg/mL)	82.5 [55.0–282.2]	229.0 [97.0–602.0]	69.5 [14.8–245.8]	<0.001	+0.880 [0.688, 1.000]

All comparisons by Wilcoxon signed-rank test applied to the within-patient differences. Effect size is the matched-pairs rank-biserial correlation; positive values indicate predominantly higher postoperative measurements and negative values predominantly lower. Confidence intervals are percentile bootstrap with 1000 resamples. The six comparisons form a predefined physiological panel and the *p*-values shown are nominal. Under a Holm–Bonferroni sensitivity check across the six comparisons, all remained significant (largest adjusted *p* = 0.015, for TAPSE). RV–PA coupling is TAPSE, converted to millimeters, divided by PASP, and analyzed as a continuous variable. Echocardiography was performed on the first postoperative day; NT-proBNP was sampled at 24 h. IQR, Interquartile range; LVEF, left ventricular ejection fraction; TAPSE, tricuspid annular plane systolic excursion; PASP, systolic pulmonary artery pressure; TRV, tricuspid regurgitant velocity; RV–PA, right ventricular to pulmonary arterial.

**Table 3:** Exploratory associations between perioperative changes, NT-proBNP and postoperative complications (N = 40).

Predictor	Complication, Median [IQR]	No Complication, Median [IQR]	*p*	Rank-Biserial r [95% CI]	AUC [95% CI]
Panel A. Any postoperative complication (16 events versus 24)					
Change in LVEF (%)	−3.0 [−5.0, −0.8]	−1.0 [−4.2–0.0]	0.235	−0.221 [−0.560, 0.148]	0.389 [0.204, 0.570]
Change in TAPSE (cm)	−0.10 [−0.35, −0.05]	−0.10 [−0.20–0.10]	0.408	−0.156 [−0.518, 0.183]	0.422 [0.249, 0.607]
Change in PASP (mmHg)	3.0 [1.8–7.5]	6.5 [0.8–10.0]	0.628	−0.094 [−0.440, 0.281]	0.453 [0.271, 0.633]
Change in TRV max (m/s)	0.15 [0.10–0.34]	0.10 [−0.02–0.60]	0.989	+0.005 [−0.385, 0.370]	0.503 [0.328, 0.689]
Change in RV–PA coupling	−0.081 [−0.318, −0.051]	−0.132 [−0.226, −0.017]	0.629	−0.094 [−0.479, 0.250]	0.453 [0.277, 0.658]
NT-proBNP (preoperative) (pg/mL)	105.5 [54.5–463.2]	77.0 [58.5–166.5]	0.571	+0.109 [−0.305, 0.490]	0.555 [0.352, 0.749]
NT-proBNP 24 h (pg/mL)	473.0 [194.2–1024.0]	157.0 [89.8–483.0]	0.015	+0.464 [0.104, 0.740]	0.732 [0.544, 0.875]
Change in NT-proBNP (pg/mL)	225.0 [45.0–729.8]	44.5 [10.2–179.0]	0.047	+0.378 [0.018, 0.688]	0.689 [0.503, 0.854]
Panel B. Cardiopulmonary complication (7 events versus 33)					
Change in LVEF (%)	−5.0 [−5.0, −2.0]	−2.0 [−5.0–0.0]	0.126	−0.368 [−0.749, 0.061]	0.316 [0.112, 0.520]
Change in TAPSE (cm)	−0.10 [−0.40–0.00]	−0.10 [−0.20–0.10]	0.639	−0.117 [−0.632, 0.437]	0.442 [0.181, 0.760]
Change in PASP (mmHg)	3.0 [1.5–9.0]	4.0 [1.0–10.0]	0.957	−0.017 [−0.498, 0.481]	0.491 [0.238, 0.760]
Change in TRV max (m/s)	0.22 [0.00–0.30]	0.10 [0.03–0.50]	0.886	−0.039 [−0.533, 0.481]	0.481 [0.176, 0.745]
Change in RV–PA coupling	−0.092 [−0.253, −0.043]	−0.125 [−0.246, −0.030]	0.972	−0.013 [−0.515, 0.481]	0.494 [0.243, 0.781]
NT-proBNP (preoperative) (pg/mL)	55.0 [48.0–462.0]	84.0 [57.0–201.0]	0.749	−0.082 [−0.675, 0.502]	0.459 [0.149, 0.816]
NT-proBNP 24 h (pg/mL)	929.0 [418.5–1592.5]	190.0 [93.0–501.0]	0.004	+0.701 [0.368, 0.939]	0.851 [0.681, 0.983]
Change in NT-proBNP (pg/mL)	650.0 [225.0–1546.5]	52.0 [14.0–185.0]	0.019	+0.576 [0.065, 0.957]	0.788 [0.461, 0.989]

All comparisons by Mann–Whitney U test; positive rank-biserial values indicate higher measurements in the complication group. The AUC is reported with each predictor in its recorded direction, without sign inversion; values below 0.50 therefore indicate that lower measurements accompanied complications. No cut-offs, sensitivities or specificities were derived. All analyses are unadjusted and exploratory, and *p*-values are nominal and uncorrected for multiplicity. Confidence intervals are percentile bootstrap with 1000 resamples.

## Data Availability

The data are encapsulated within this article. Further details can be obtained upon request from either the primary author or the corresponding authors.

## Supplementary Materials

The following supporting information can be downloaded at https://www.mdpi.com/article/10.3390/jcm15187273/s1, Table S1: Perioperative changes in surgical approach; Table S2: Perioperative changes in resection side; Table S3: Perioperative changes in lobe of resection; Table S4: Normality diagnostics for the within-patient differences; Table S5: Distribution of the RV–PA coupling ratio relative to published reference values; Table S6: Sensitivity analysis and detectable effects; Figure S1: Histograms and quantile–quantile plots of the within-patient differences; Figure S2: Perioperative changes in lobe of resection; Figure S3: Minimum detectable standardized paired effect versus paired sample size.
