# Supplementary material for: Early Postoperative Echocardiographic and Natriuretic Peptide Changes Following Anatomical Lung Resection: A Single-Center Cohort Study
Source: J Clin Med. 2026 Sep 18;15(18):7273. doi: 10.3390/jcm15187273 (PMC13607337; doi:10.3390/jcm15187273)

Journal of Clinical Medicine

MDPI

Supplementary Materials

Table S1: Perioperative changes in surgical approach. No a priori direction.

Parameter	ΔPASP (mmHg)	ΔTRV max (m/s)	ΔTAPSE (cm)	ΔLVEF (%)
n VATS	9	9	9	9
VATS median [IQR]	10.0 [3.0–10.0]	0.20 [0.0–0.70]	0.10 [-0.10–0.10]	-3.0 [-5.0–0.0]
n Open	31	31	31	31
Open median [IQR]	4.0 [1.0–8.5]	0.10 [0.06–0.45]	-0.10 [-0.35–0.00]	-2.0 [-5.0–0.0]
Diff. (VATS − Open)	6	0.1	0.2	-1
Mann–Whitney U	158.5	152	176.5	133.5
p-value	0.548	0.696	0.23	0.855
Rank-biserial r	0.136	0.09	0.265	-0.043
r 95% CI (bootstrap)	-0.323 to 0.534	-0.401 to 0.523	-0.111 to 0.627	-0.495 to 0.384
Observed direction	No a priori direction	No a priori direction	No a priori direction	No a priori direction

VATS n=9 against Open n=31. This exploratory comparison involves small, imbalanced groups and yields imprecise estimates; p-values are nominal and descriptive.

No a priori direction is claimed for surgical approach, regardless of tier.

Orientation: Rank-biserial r = 2*U1/(n1*n2) - 1 with group 1 = VATS, so r > 0 means the VATS group has the more positive deltas. The difference column is oriented the same way (VATS − Open).

CI = Percentile bootstrap, 1000 resamples, seed 42, stratified within group so both group sizes are preserved.

Table S2: Perioperative changes in resection side.

Parameter	ΔPASP (mmHg)	ΔTRV max (m/s)	ΔTAPSE (cm)	ΔLVEF (%)
n Right	25	25	25	25
Right median [IQR]	3.0 [-1.0–10.0]	0.10 [-0.10–0.46]	-0.10 [-0.10–0.10]	-1.0 [-5.0–0.0]
n Left	15	15	15	15
Left median [IQR]	5.0 [2.5–9.5]	0.10 [0.10–0.41]	-0.10 [-0.45–-0.10]	-3.0 [-5.0–-1.0]
Diff. (Right − Left)	-2	0	0	2
Mann–Whitney U	149	160	240	223.5
p-value	0.287	0.447	0.14	0.31
Rank-biserial r	-0.205	-0.147	0.28	0.192
r 95% CI (bootstrap)	-0.531 to 0.184	-0.483 to 0.235	-0.072 to 0.621	-0.125 to 0.523
Observed direction	Left larger	Tie	No a priori direction	No a priori direction

Right n=25 against Left n=15. This exploratory comparison involves small, imbalanced groups and yields imprecise estimates; p-values are nominal and descriptive.

No directional hypothesis was prespecified for resection side. As the right lung carries roughly 55% of the pulmonary vascular bed, right-sided resection loads the remaining circulation more and is expected to show the larger ΔPASP and ΔTRV max. Groups are oriented Right first so a positive difference matches the hypothesis. A greater response after right-sided resection is physiologically plausible, but this interpretation is post hoc and is reported descriptively.

Direction is reported, not tested one-sided: every test here is two-sided and all four deltas are reported regardless of significance.
Side coding: 0 = Left, 1 = Right.
Orientation: Rank-biserial r = 2*U1/(n1*n2) - 1 with group 1 = Right, so r > 0 means the Right group has the more positive deltas. The difference column is oriented the same way (Right − Left).
CI = percentile bootstrap, 1000 resamples, seed 42, stratified within group so both group sizes are preserved.

Table S3: Perioperative changes in lobe of resection.

Parameter	Lower lobe (n = 14), median [IQR]	Upper lobe (n = 21), median [IQR]	Difference (lower minus upper)	p	Rank-biserial r [95% CI]
Primary parameters (a priori expectation: larger increase after lower lobe resection)					
Change in PASP (mmHg)	5.0 [2.2, 13.8]	3.0 [0.0, 10.0]	+2.0	0.389	+0.177 [-0.224, 0.555]
Change in TRV max (m/s)	0.24 [0.10, 0.58]	0.10 [-0.10, 0.46]	+0.14	0.285	+0.218 [-0.160, 0.616]
Secondary parameters (no a priori direction)					
Change in TAPSE (cm)	-0.10 [-0.47, 0.05]	-0.10 [-0.20, 0.10]	0.00	0.441	-0.156 [-0.534, 0.269]
Change in LVEF (%)	-2.0 [-4.5, 0.0]	-3.0 [-5.0, 0.0]	+1.0	0.784	-0.058 [-0.435, 0.337]

Exploratory subgroup comparison. Middle lobe resections and bilobectomies were not tested and are reported in the notes below.
Comparisons by Mann–Whitney U test, two-sided. Effect size is the rank-biserial correlation oriented so that positive values indicate more positive changes in the lower lobe group. Confidence intervals are percentile bootstrap with 1000 resamples, stratified within group.
Lobe assignment: The resection variable records lobe positions: right upper, right middle, right lower, left upper, left lower, and bilobectomy. The upper group therefore comprises right and left upper lobe resections (n = 21) and the lower group right and left lower lobe resections (n = 14). All procedures were lobectomies or bilobectomies; no pneumonectomy or sublobar resection was performed.
A priori expectation: Because the lower lobes receive a greater share of pulmonary perfusion, larger increases in PASP and in TRV max were anticipated after lower lobe resection. Both primary parameters moved in the anticipated direction, but neither reached statistical significance and both confidence intervals include zero; the direction is therefore consistent with the expectation but the comparison does not support it. All four parameters are reported regardless of significance.
Groups not tested: Middle lobe resection (n = 2) and bilobectomy (n = 3) were not tested: two and three observations cannot support inference, and bilobectomy is a different operation from a single-lobe resection. Individual values are given here rather than medians. Middle lobe, change in PASP of 8.0 and 11.0 mmHg; change in TRV max of 0.10 and 0.30 m/s; change in TAPSE of -0.10 and -0.20 cm; change in LVEF of -5.0 and 0.0 percentage points. Bilobectomy, change in PASP of 1.0, -3.0 and 1.0 mmHg; change in TRV max of -0.10, 0.03 and 0.22 m/s; change in TAPSE of 0.10, 0.20 and -0.90 cm; change in LVEF of -3.0, 0.0 and -5.0 percentage points.
This is an exploratory subgroup analysis. The p-values are nominal and are not corrected for multiplicity across the four parameters or across the three subgroup contrasts examined, and should be read as hypothesis-generating.

Abbreviations: IQR, Interquartile range; LVEF, left ventricular ejection fraction; PASP, systolic pulmonary artery pressure; TAPSE, tricuspid annular plane systolic excursion; TRV, tricuspid regurgitant velocity.

Table S4: Normality diagnostics for the within-patient differences.

Parameter	LVEF (%)	TAPSE (cm)	PASP (mmHg)	TRV max (m/s)	RV–PA coupling (mm/mmHg)	NT-proBNP (pg/mL)
n pairs	40	40	40	40	40	40
n zero differences	12	1	1	1	0	0
Difference mean (SD)	-2.0 (3.9)	-0.16 (0.41)	6.0 (7.4)	0.24 (0.35)	-0.163 (0.254)	319.0 (721.9)
Difference median [IQR]	-2.0 [-5.0–0.0]	-0.10 [-0.23–0.10]	4.0 [1.0–10.0]	0.10 [0.02–0.47]	-0.108 [-0.256–-0.032]	69.5 [14.8–245.8]
Difference min	-10	-1.55	-4	-0.4	-1.008	-237
Difference max	12	0.5	22	1.2	0.264	3996
Shapiro–Wilk W (differences)	0.8681	0.8422	0.9246	0.9399	0.9155	0.5065
Shapiro–Wilk p (differences)	<0.001	<0.001	0.011	0.034	0.006	<0.001
Differences normal?	No	no	no	no	no	no
Test used	Wilcoxon signed-rank	Wilcoxon signed-rank	Wilcoxon signed-rank	Wilcoxon signed-rank	Wilcoxon signed-rank	Wilcoxon signed-rank
Wilcoxon statistic	60	217.5	89.5	122.5	131	49
Wilcoxon p	0.001	0.015	<0.001	<0.001	<0.001	<0.001
Rank-biserial r	-0.704	-0.442	0.771	0.686	-0.68	0.88
r 95% CI (bootstrap)	-0.992 to -0.332	-0.730 to -0.124	0.529 to 0.933	0.427 to 0.887	-0.895 to -0.414	0.688 to 1.000
pingouin RBC (cross-check)	-0.704	-0.442	0.771	0.686	-0.68	0.88

Shapiro–Wilk is applied to the within-patient differences (post - pre), not to the pre/post marginals. Wilcoxon signed-rank is reported for every parameter regardless of the Shapiro result, so the paired analysis is uniformly non-parametric; the Shapiro column documents whether that choice was necessary or merely conservative. Rank-biserial r replaces Cohen's d. r > 0 means post-op values are predominantly higher than pre-op. CI = Percentile bootstrap, 1000 resamples, seed 42, resampling complete pre/post pairs. pingouin's RBC is a cross-check only; a sign difference reflects its own orientation convention, not a discrepancy in the test.

Table S5: Distribution of the RV–PA coupling ratio relative to published reference values.

Reference (mm/mmHg)	n pre-op above	n pre-op at or below	n pre-op evaluable	n post-op above	n post-op at or below	n post-op evaluable
0.31	39	1	40	38	2	40
0.67	23	17	40	10	30	40

These counts locate the cohort relative to two literature reference values for the TAPSE/PASP ratio. They are not used to classify patients as 'preserved' or 'impaired' coupling; no group is formed from them; no test is run on them, and no conclusion about preserved vs impaired coupling is drawn anywhere in this analysis.

Table S6: Sensitivity analysis and detectable effects.

Panel A. Paired within-patient comparisons

Quantity	Value
Paired observations (n)	40
Significance level (two-sided)	0.05
Target power	0.80
Minimum detectable dz, paired t-test	0.454
Asymptotic relative efficiency applied (Wilcoxon)	0.955
Effective n for the Wilcoxon signed-rank test	38.2
Minimum detectable dz, Wilcoxon signed-rank test	0.465

Panel B. Between-group comparisons by complication outcome

Group sizes	Basis	Minimum detectable d	Equivalent AUC	Effective n
Any postoperative complication (16 events of 40)				
16 vs 24 (observed)	Normal theory	0.904	0.739	38.4
16 vs 24 (observed)	Mann–Whitney, ARE applied	0.925	0.744	36.7
20 vs 20 (hypothetical)	Mann–Whitney, ARE applied	0.907	0.739	38.2
Cardiopulmonary complication (7 events of 40)				
7 vs 33 (observed)	Normal theory	1.166	0.795	23.1
7 vs 33 (observed)	Mann–Whitney, ARE applied	1.193	0.801	22.1
20 vs 20 (hypothetical)	Mann–Whitney, ARE applied	0.907	0.739	38.2

No a priori sample size calculation was performed, and achieved power is not reported. This table instead characterizes the magnitude of effect the study was able to detect, given the number of patients and events actually available. These quantities are derived from the available sample size, realized event/non-event counts, significance level and target power; none is computed from an observed predictor effect size.

These are detectable effects, not achieved or post hoc power. Nothing in this table is computed from an observed effect size. The question addressed is how large an effect would have had to be before this study could detect it, at a significance level of 0.05 (two-sided) and 80% power.

Group sizes are the observed event and non-event counts throughout. Complication status is a realized property of the cohort rather than an allocated design factor, so assuming balanced groups would describe a study that was never conducted and would overstate what this one could detect.

The conversion from a standardized difference to the AUC scale uses the normal-scores approximation; AUC equals the standard normal distribution function evaluated at d divided by the square root of two. It is a translation of the same quantity onto the scale on which discrimination is reported, not a separately derived result, and is exact only when the predictor is normally distributed in both groups.

Where the asymptotic relative efficiency of 0.955 is applied, it is used as an effective sample size adjustment. This raises the detectable effect and is therefore the conservative direction.

Panel A describes detectability for a single paired comparison at nominal two-sided $\alpha = 0.05$ and does not incorporate the Holm family-wise sensitivity procedure. The dz is the standardized within-patient effect, defined as the mean of the paired differences divided by their standard deviation, solved from the noncentral t distribution. At a fixed sample size this quantity is identical for every parameter, so no per-parameter rows are given.

Panel B: Group sizes are the observed numbers of patients with and without each outcome. The balanced 20 versus 20 rows are hypothetical and are included only to quantify the cost of the observed imbalance; they do not describe this study. For the cardiopulmonary outcome the imbalance reduces the effective sample size from 40 to approximately 22 patients and raises the detectable AUC from 0.739 to 0.801.

Figure S1: Histograms and quantile–quantile plots of the within-patient differences.

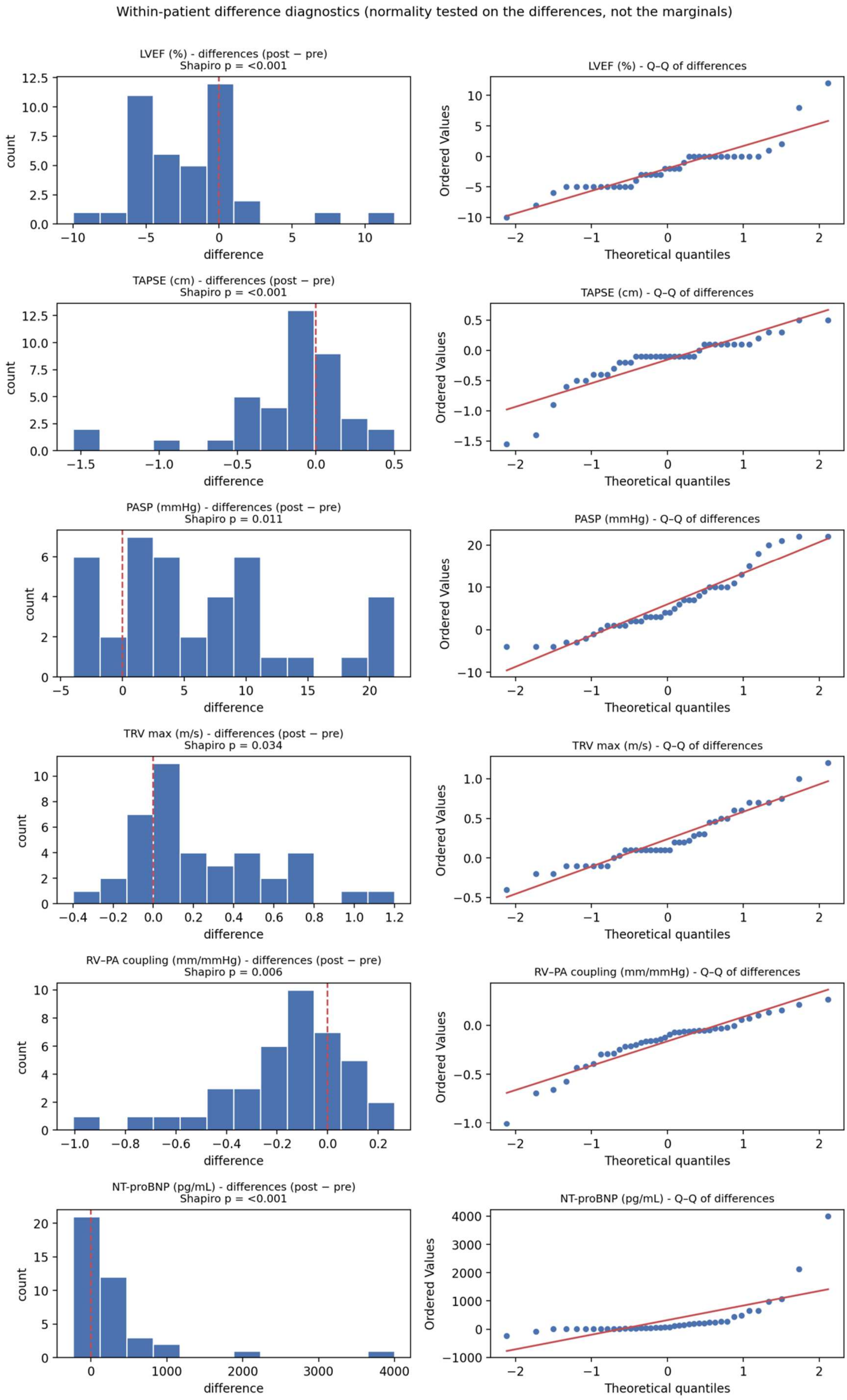

Figure S2: Perioperative changes in lobe of resection
(Middle and bilobectomy shown in grey, not tested).

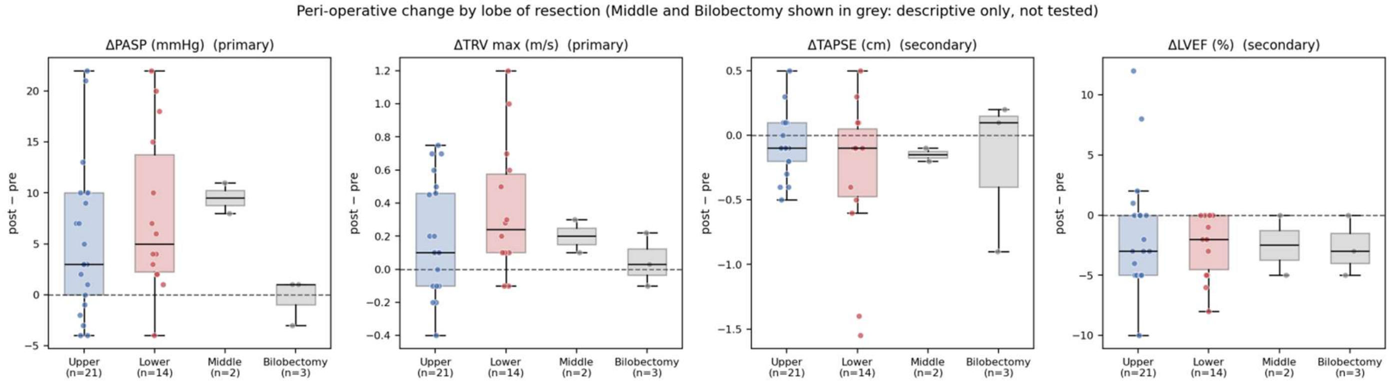

Figure S3: Minimum detectable standardized paired effect versus paired sample size.

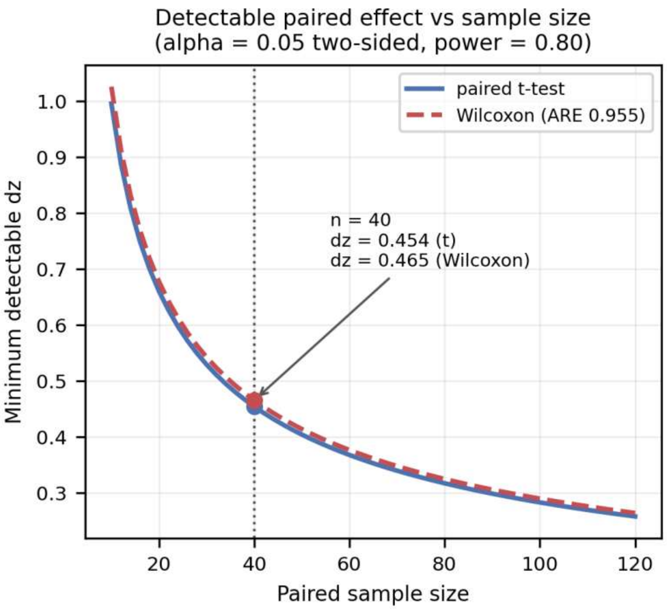

Supplement: Supplementary file 1 [file jcm-15-07273-s001.zip › jcm-4558771-supplementary.pdf]
